# Telestroke strategies to enhance acute stroke management in rural settings: A systematic review and meta‐analysis

**DOI:** 10.1002/brb3.1787

**Published:** 2020-08-18

**Authors:** Gilbert Lazarus, Affan Priyambodo Permana, Setyo Widi Nugroho, Jessica Audrey, Davin Nathan Wijaya, Indah Suci Widyahening

**Affiliations:** ^1^ Faculty of Medicine Universitas Indonesia Jakarta Indonesia; ^2^ Department of Neurosurgery Faculty of Medicine Universitas Indonesia, Dr. Cipto Mangunkusumo National General Hospital Jakarta Indonesia; ^3^ Department of Community Medicine Faculty of Medicine, Universitas Indonesia Jakarta Indonesia

**Keywords:** emergency care, rural health, stroke, teleconsultation, telemedicine

## Abstract

**Background:**

The potential of telestroke implementation in resource‐limited areas has yet to be systematically evaluated. This study aims to investigate the implementation of telestroke on acute stroke care in rural areas.

**Methods:**

Eligible studies published up to November 2019 were included in this study. Randomized trials were further evaluated for risk of bias with Cochrane RoB 2, while nonrandomized studies with ROBINS‐I tool. Random effects model was utilized to estimate effect sizes, and the certainty of evidence was assessed using the Grading of Recommendations Assessment, Development, and Evaluation (GRADE) tool.

**Results:**

The search yielded 19 studies involving a total of 28,496 subjects, comprising of prehospital and in‐hospital telestroke interventions in the form of mobile stroke units and hub‐and‐spoke hospitals network, respectively. Telestroke successfully increased the proportion of patients treated ≤3 hr (OR 2.15; 95% CI 1.37–3.40; *I* ^2^ = 0%) and better three‐month functional outcome (OR 1.29; 95% CI 1.01–1.63; *I* ^2^ = 44%) without increasing symptomatic intracranial hemorrhage rate (OR 1.27; 0.65–2.49; *I* ^2^ = 0%). Furthermore, telestroke was also associated with shorter onset‐to‐treatment time (mean difference −27.97 min; 95% CI −35.51, −20.42; *I* ^2^ = 63%) and lower in‐hospital mortality rate (OR 0.67; 95% CI 0.52–0.87; *I* ^2^ = 0%). GRADE assessments yielded low‐to‐moderate certainty of body evidences.

**Conclusion:**

Telestroke implementation in rural areas was associated with better clinical outcomes as compared to usual care. Its integration in both prehospital and in‐hospital settings could help optimize emergency stroke approach. Further studies with higher‐level evidence are needed to confirm these findings.

## Graphical abstract

Telestroke implementation in rural areas was associated with better clinical outcomes as compared to usual care, evident in the increased proportion of patients treated within 3 hr and better three‐month functional outcome without increasing symptomatic intracranial hemorrhage rate. Furthermore, telestroke was also associated with shorter onset‐to‐treatment time and lower in‐hospital mortality rate. Its integration in both prehospital and in‐hospital settings could help optimize emergency stroke approach.

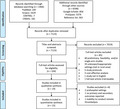

## 1 INTRODUCTION

Stroke is the second leading cause of mortality and third leading cause of disability worldwide (Johnson, Onuma, Owolabi, & Sachdev, 2016). This alarming evidence is aggravated by the fact that about 87% of stroke‐related deaths occurred in low‐ and middle‐income countries where 80% of the population reside in rural areas (Joubert et al., 2008), which is an exclusion term of urbanized area generally characterized by low population density and distant urban facilities (Hart, Larson, & Lishner, 2005). The vulnerability of rural populations is evident in the lack of resources and predominant treatment delays (Kapral et al., 2019).

Despite the establishment of systemic thrombolysis in treating stroke, its mortality remains high, mainly attributing to the delayed presentation of patients (Al Khathaami, Mohammad, Alibrahim, & Jradi, 2018). With the advent of technologies, telestroke arises as a promising intervention capable of providing treatment to stroke victims in rural communities. Although several previous meta‐analyses have proven the safety and efficacy of telestroke (Zhai, Zhu, Hou, Sun, & Zhao, 2015; Kepplinger et al., 2016; Baratloo et al., 2018; McDermott, Skolarus, & Burke, 2019), no study has yet to investigate the use of this novel approach in resource‐limited settings. Hence, this systematic review and meta‐analysis were conducted to critically evaluate the use of telestroke on acute stroke management in rural areas.

## 2 MATERIALS AND METHODS

A systematic review was conducted based on the Cochrane Handbook for Systematic Reviews of Intervention ver. 5.1.0 (Higgins & Green, 2011) and reported according to the Preferred Reporting Items for Systematic Reviews and Meta‐Analyses (PRISMA) statement (Moher, Liberati, Tetzlaff, & Altman, 2009).

### 2.1 Search strategy

**FIGURE 1:**
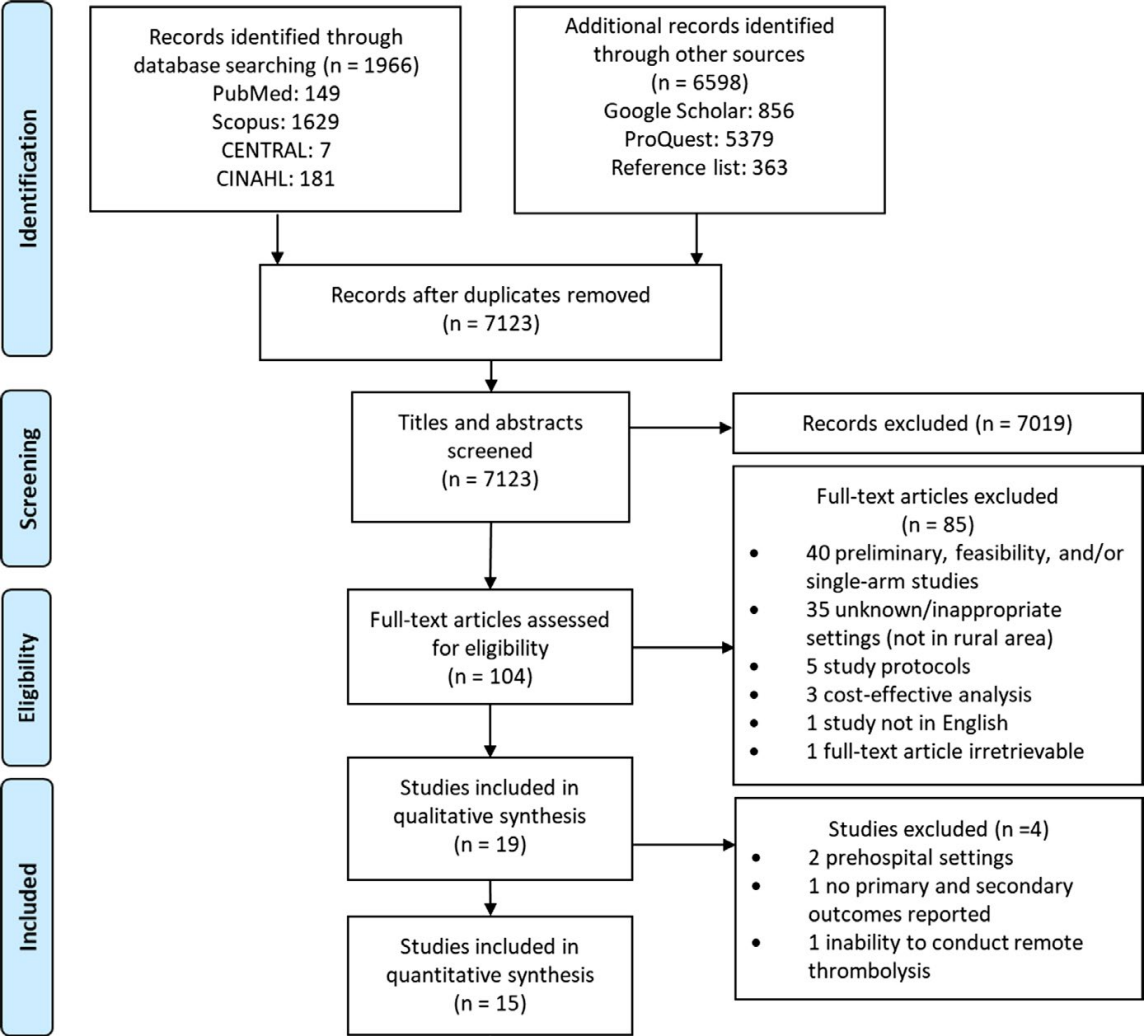
Preferred reporting items for systematic reviews and meta‐analyses (PRISMA) flow diagram. CENTRAL, Cochrane Central Register of Controlled Trials; CINAHL, Cumulative Index to Nursing and Allied Health Literature

Relevant studies from PubMed, Scopus, Cochrane Controlled Register of Trials (CENTRAL), and CINAHL databases published up to November 2019 were screened using keywords listed on Table S1. Additionally, Google Scholar and ProQuest databases were screened for grey literatures. Manual searches were performed by hand‐searching reference lists from included studies and reviews. Literature searches were conducted in pair (GL and JA), and any title and abstracts judged potentially eligible by either reviewer were retrieved for full‐text assessment. Any discrepancies were resolved by a third reviewer (AP). Details on the literature search process are shown on Figure 1.

### 2.2 Study eligibility criteria

Inclusion criteria were set to filter interventional studies involving acute stroke patients receiving telestroke care in rural or nonurban area. Interventions were implementation of telestroke in comparison with any other interventions. Any outcomes were incorporated, including treatment times and rates, mortality rates, and functional outcome rates. Criteria for exclusion were as follows: (a) irretrievable full‐text articles, (b) preliminary, feasibility, or single‐arm studies, and (c) articles not in English.

In the case of studies with unknown settings (e.g., when the authors did not explicitly state rural/nonurban settings), corresponding authors were contacted to obtain this information. When no response was given by the authors, the article was excluded from this review. Telestroke is defined as the use of technology in providing acute stroke care to overcome the lack of expertise and resources, which may be applied as part of prehospital and/or in‐hospital services (Demaerschalk et al., 2017).

### 2.3 Data extraction and risk‐of‐bias assessment

Essential data from studies were extracted, generally classified as: (a) author and year of publication; (b) study characteristics (e.g., study design, location, settings, technology utilized in telestroke arm, controls designed in the study); (c) subject characteristics (e.g., sample size, mean age, and proportion of male populations); and (d) type of outcomes and its effect sizes.

Primary outcomes include intravenous thrombolysis (IVT) rate and onset‐to‐treatment time (OTT). Secondary outcomes consist of number of patients treated within 4.5 hr—as per guideline (Powers et al., 2018), 3‐month functional outcome rate—defined as modified Rankin scale (mRS) score ≤2 (Sulter, Steen, & De Keyser, 1999), and safety outcomes (i.e., in‐hospital mortality and symptomatic intracranial hemorrhage (sICH) post‐IVT. In addition, any other reported outcomes were also extracted.

Risk of bias of each included randomized study was assessed using Revised Cochrane risk‐of‐bias tool for randomized trials (RoB 2) (Sterne et al., 2019), while nonrandomized studies with Risk of Bias in Non‐randomized Studies of Intervention (ROBINS‐I) (Sterne et al., 2016) tool. Since ROBINS‐I checklist specifically developed for pre–post‐studies are yet to be published, the confounding bias of these studies was judged serious due to observed general trend of reduced OTT and increased IVT administration (Muller‐Barna et al., 2014). Risk‐of‐bias assessments were conducted by two reviewers independently (GL and DNW), and discrepancies were resolved by consensus between a third reviewer (AP), according to a standardized protocol. Figure S1 and Table S2 provide details of risk of bias of included randomized and nonrandomized studies, respectively (Sterne et al., 2019).

Lastly, the overall quality of evidence was appraised using the Grading of Recommendations Assessment, Development, and Evaluation (GRADE) approach, where the certainty of the body evidences was graded as high, moderate, low, and very low (Guyatt et al., 2008).

### 2.4 Statistical analysis

Continuous data were pooled as mean ± *SD*. When mean and *SD* were unavailable, corresponding authors of the respective study were contacted. There were at least 10 contacts attempted to obtain missing data or confirm the stroke centers certification or rural populations. In the case of unresponsive authors or unavailable data, mean and *SD* were calculated from the median, range, interquartile range (IQR), or sample size (Wan, Wang, Liu, & Tong, 2014). In the case where 2 or more studies involved overlapping populations (Audebert, Kukla, et al., 2006; Audebert, Schenkel, Heuschmann, Bogdahn, & Haberl, 2006; Audebert et al., 2009; Muller‐Barna et al., 2014; Schwab et al., 2007), analysis was conducted on studies which had bigger sample size.

Statistical analysis was performed using the Review Manager 5.3 (The Nordic Cochrane Centre, The Cochrane Collaboration, 2014, Copenhagen), while additional sensitivity analysis using MetaXL software ver 5.3. (www.epigear.com). As clinical heterogeneity was expected, a random effect model was used. Heterogeneity between studies was investigated with Cochran *Q* test, chi‐squared statistics, and *I* ^2^ value, which explains the degree of variability between studies due to true heterogeneity rather than chance. *I* ^2^ values were classified as no (0%–25%), low (25%–50%), moderate (50%–75%), and high (>75%) heterogeneity. Dichotomous outcomes were presented in odds ratios (ORs) using the Mantel–Haenszel method, while continuous data in mean difference (MD) using the inverse variance weighing. A *p* value of ≤.05 is considered as statistically significant. A priori, we prespecified subgroup and sensitivity analyses only for primary outcomes. Subgroup analysis was performed to identify any difference of outcomes when categorized by control group, while sensitivity analysis was performed by leave‐one‐out method. Funnel plots were generated to evaluate potential publication bias when the number of studies was adequate, with symmetry evaluated qualitatively by visual inspection and quantitatively by Egger's test.

## 3 RESULTS

### 3.1 Study selection and characteristics

Figure 1 provides the details on the literature screening process for included studies in this systematic review. The initial search yielded 8,564 relevant studies. Subsequently, 1,441 articles were deduplicated and 7,019 were excluded after title and abstracts screening. Hence, 104 articles were retrieved for full‐text review, of which 85 were excluded. As a result, 19 studies with a pooled total subject of 28,496 patients were included in this review, comprising of four randomized studies, 12 nonrandomized studies, and three pre–post‐studies. Among these studies, two studies evaluated the use of prehospital telestroke technology incorporated into an ambulance (i.e., mobile stroke unit [MSU]), while the other 17 established a hub‐and‐spoke hospital network (classified as in‐hospital). Quantitative analysis was conducted only for outcomes following the implementation of in‐hospital telestroke, as evidence on the use of prehospital telestroke was limited. A total of 14 studies were analyzed quantitatively—where two (Helwig et al., 2019; Walter et al., 2012) were excluded due to prehospital settings, one (Audebert et al., 2009) due to no primary and secondary outcomes reported, and one (Dharmasaroja, Muengtaweepongsa, & Kommarkg, 2010) due to inappropriate study procedure as remote thrombolysis was not available; hence, patients eligible for treatment were referred to stroke center, which surely confirmed the presence of longer stroke time metrics.

The included studies were published between 2000 and 2019. Ten studies were conducted in Europe (eight in Germany, one in Spain, and one in United Kingdom), seven in North America (six in the United States of America and one in Canada), and two in Asia‐Pacific (Australia and Thailand). Risk‐of‐bias assessment of randomized studies resulted in one study with low risk, two with unclear risk, and an another one with high risk, while risk‐of‐bias assessment of nonrandomized studies resulted in low risk for two studies, moderate risk for eight studies, and serious risk for the other five. With regard to the telemedical approaches, videoconference was utilized in 15 studies, telephone in eight studies, and both interventions were implemented in four studies. Telestroke interventions in rural settings were classified to two main categories: as part of prehospital and as part of in‐hospital management (Table S3).

### 3.2 Prehospital settings

Prehospital telestroke service in rural areas emerged in the form of MSUs. MSU is proven capable to provide better diagnosis, as shown by higher sensitivity, specificity, positive predictive value, and negative predictive value (100% versus 35.3%, 86.1%, 54.5%, 73.8%, respectively; Table S4) when compared to control. Furthermore, substantially higher triage accuracy was observed in MSU arm when compared to conventional emergency medical service (EMS; 100% versus 69.8%, *p* < .001). In addition to higher triage and diagnosis accuracies, the implementation of MSU in stroke networks also resulted in shorter alarm‐to‐needle time (−34.8 min, *p* < .001 (Helwig et al., 2019); and −43 min, *p* < .001 (Walter et al., 2012)) as well as time from call to imaging‐based triage and therapy decision (−535.4 min, *p* = .009 (Helwig et al., 2019); and −41 min, *p* < .001 (Walter et al., 2012)).

### 3.3 In‐hospital settings

Telestroke in the in‐hospital management of acute stroke patients arose in the form of hub‐and‐spoke hospitals networks which allowed emergency physicians at spoke hospitals to perform remote thrombolysis by the guide of neurologists at the hub sites. Table 1 provides summary of findings of outcomes on in‐hospital management, as assessed with the GRADE approach. We discovered that telestroke was likely to increase the number of patients treated within the golden window (≤3 hr), as seen by moderate certainty of evidence. Furthermore, telestroke had little to no effect on IVT rate and in‐hospital mortality, while it might result in slight increase on the favorable outcome rate in 3‐month time (quality of evidence: very low). The use of telestroke was also associated with shorter OTT, while it did not increase sICH rate when compared to usual care (graded low strength of evidence). Details on GRADE assessment of evidences’ quality are shown on Table S5.

**TABLE 1:** Summary of findings

Outcomes	No of participants (studies)	Relative effect OR (95% CI)	Absolute effects per 1,000 (95% CI)		Certainty of the evidence (GRADE)
			Risk with control	Risk with telestroke	
IVT rate	7,665 (4)	2.60 (0.89 to 7.57)	49	118 (44 to 280)	⨁ Very low
OTT	8,112 (6)	–	–	MD −27.97 (−35.51 to −20.42)	⨁⨁ Low
Patients treated ≤3 hr	629 (3)	2.15 (1.37 to 3.40)	593	758 (666 to 832)	⨁⨁⨁ Moderate
In‐hospital mortality	6,919 (4)	0.67 (0.52 to 0.87)	53	36 (29 to 47)	⨁ Very low
3‐month functional outcome rate	3,854 (3)	1.29 1.01 to 1.63)	446	509 (448 to 567)	⨁ Very low
sICH	1,437 (6)	1.27 (0.65 to 2.49)	25	32 (17 to 61)	⨁⨁ Low

Abbreviations: GRADE, Grading of Recommendations Assessment, Development, and Evaluation; IVT, intravenous thrombolysis; MD, mean difference; OR, odds ratio; OTT, onset‐to‐treatment time; sICH, symptomatic intracranial hemorrhage.

#### IVT rate

**FIGURE 2:**
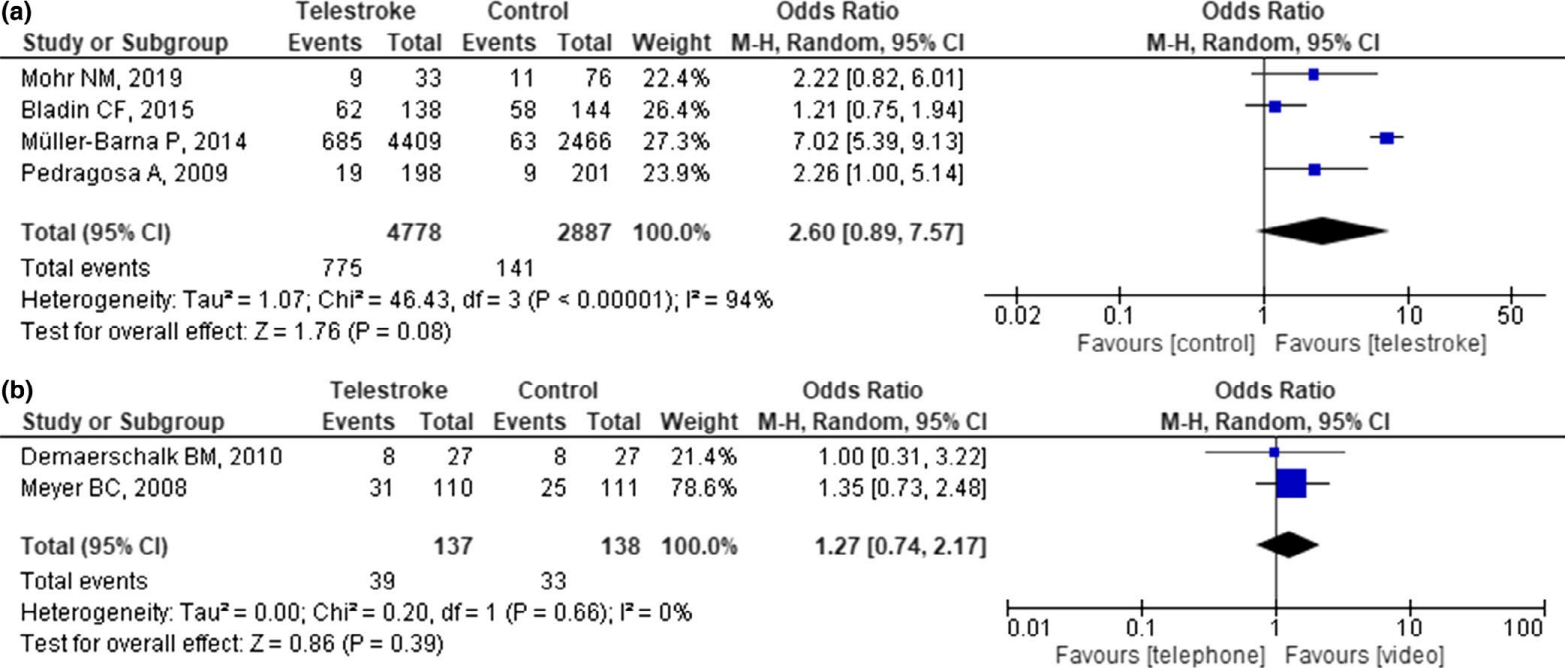
Forest plot showing the odds ratio of intravenous thrombolysis rate between (a) telestroke and stroke centers, and (b) videoconference and telephone

Overlapping populations were observed in three studies( Muller‐Barna et al., (2014); Audebert, Schenkel, et al., 2006; Audebert, Kukla, et al., 2006). The pooled results showed positive trend toward the use of telestroke, although insignificant (OR 2.60, [95% CI: 0.89–7.57], *p* = .08; Figure 2a). Furthermore, substantial heterogeneity was observed between studies (*p* < .001, *I* ^2^ = 94%), as evaluated using the random effects model. On sensitivity analysis, the exclusion of the most extreme result (Muller‐Barna et al., 2014) diminished the observed heterogeneity to 15% (*p* = .31), resulting in significant effect estimates (OR 1.56, [95% CI: 1.01, 2.41], *p* = .04; Figure S2). In addition, no superiority was detected when analysis between telemedicine approaches was performed (Figure 2b). Subgroup analysis was not performed as all studies implemented the same control group where patients are thrombolysed remotely in spoke hospitals.

#### 3.3.1 Onset‐to‐treatment time

**FIGURE 3:**
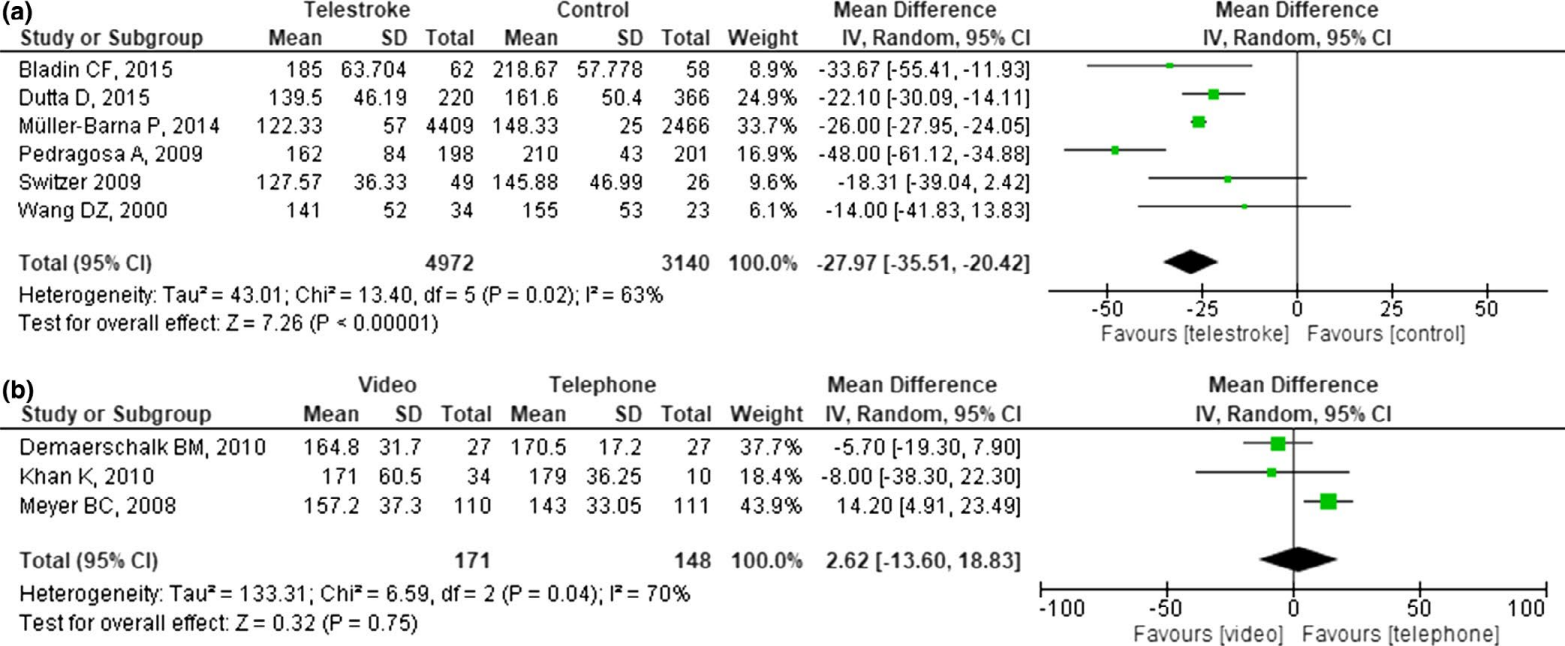
Forest plot showing the mean difference of onset‐to‐treatment time between (a) telestroke and stroke centers, and (b) videoconference and telephone

Overlapping populations were observed in two studies (Audebert, Kukla, et al., 2006; Muller‐Barna et al., 2014), of which Audebert, Kukla, et al. (2006) were excluded due to smaller sample size. Among 11 eligible studies reporting outcomes on OTT, two studies (Frey, Jahnke, Goslar, Partovi, & Flaster, 2005; Schwab et al., 2007) were excluded as only the mean times or graphic representation was reported and the authors did not respond to attempted contacts or unable to help with the data. Overall, the pooled mean difference yielded significant result with a value of −27.97 min (95% CI: −35.51, −20.42; *p* < .001), however, with moderate heterogeneity observed (*p* = .02, *I* ^2^ = 63%, Figure 3a). On subgroup analysis, three studies (7,394 patients) appointed patients transferred from spoke to hub for thrombolysis as controls. In this subgroup, telestroke was more time efficient as reduction of OTT by 35.15 min was observed (95% CI: −50.98, −19.32, *p* < .001; Figure S3), although the model yielded considerate amount of heterogeneity (*p* = .004; *I* ^2^ = 82%). When compared to walk‐in patients at stroke centers (three studies, 718 patients), telestroke implementation resulted in a reduction of OTT by 21.10 min (95% CI: −28.30, −13.89; *p* < .001) without any evidence of heterogeneity (*p* = .83, *I* ^2^ = 0%); suggesting for the noninferiority of the system.

The pooled data did not differ significantly when sensitivity analysis was conducted by removing each study one‐by‐one, indicating the robustness of our result. When the study with the most extreme result (Pedragosa et al., 2009) was excluded, heterogeneity between studies was resolved (*p* = .64, *I* ^2^ = 0%), while the effect estimate remained significant (MD −25.72 min, [95% CI: −27.60, −23.85], *p* < .001, Figure S4). No remarkable differences were observed between the use of videoconference and telephone (Figure 3b), with moderate heterogeneity observed between studies (*p* = .04, *I* ^2^ = 70%).

#### 3.3.2 Secondary outcomes

We initially searched for proportions of patients treated within 4.5 hr as per protocol. However, upon screening and extraction, we discovered that the included studies used various treatment windows, with the proportion of patients treated ≤3 hr being the most reported outcomes (i.e., four studies Audebert, Kukla, et al., 2006; Pedragosa et al., 2009; Switzer et al., 2009; Wiborg & Widder, 2003). Hence, we decided to pool the proportion of patients treated within 3 hr instead of 4.5 hr. Overlapping populations were observed in two studies (Audebert, Kukla, et al., 2006; Schwab et al., 2007) in outcome on patients treated ≤3 hr, two (Audebert, Schenkel, et al., 2006; Schwab et al., 2007) in 3‐month favorable outcomes rate, and three (Audebert, Kukla, et al., 2006; Audebert, Schenkel, et al., 2006; Muller‐Barna et al., 2014) in in‐hospital mortality rate—all of which were excluded except for studies with largest sample size on each outcome.

**FIGURE 4:**
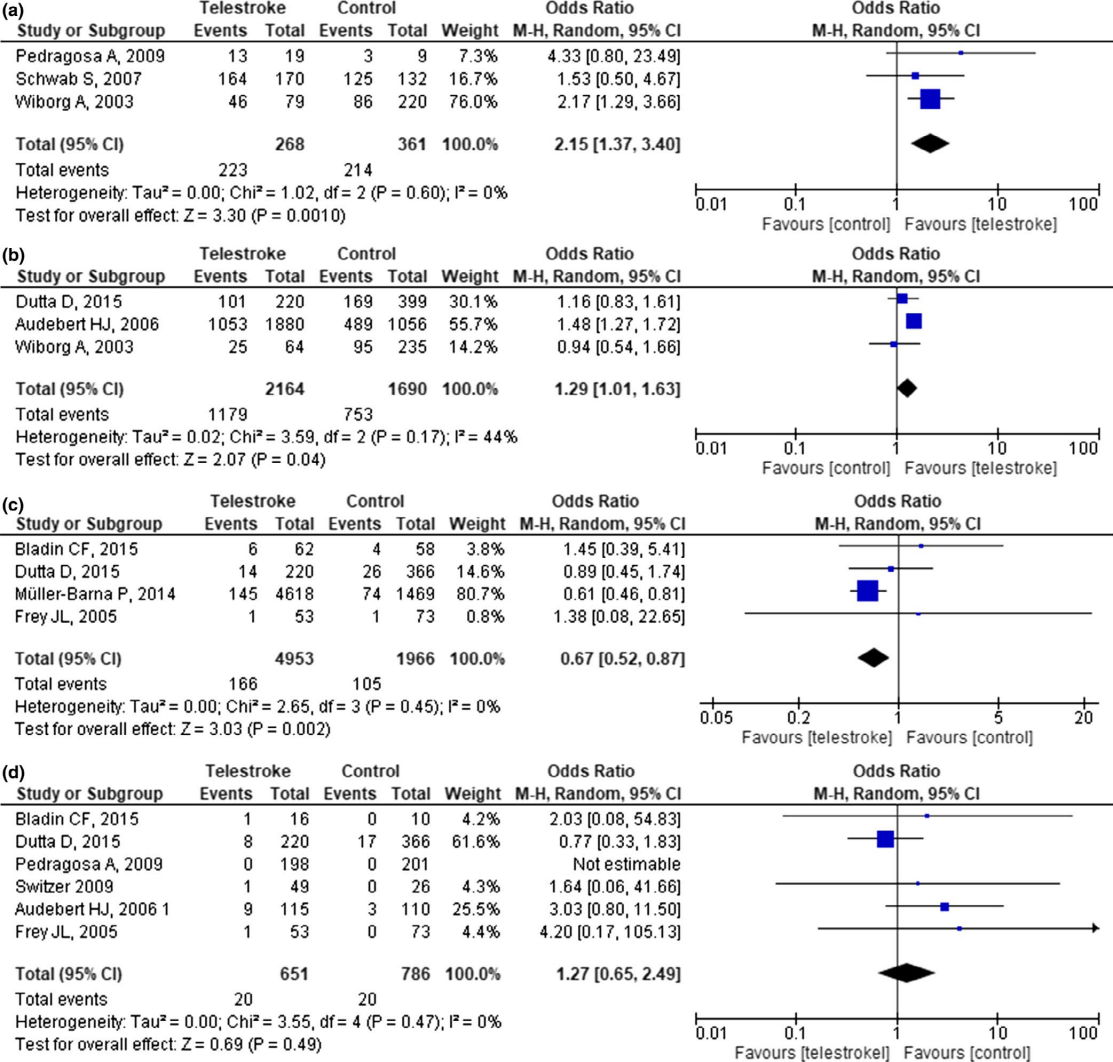
Forest plot showing the odds ratio between telestroke and stroke centers for (a) patients treated ≤3 hr, (b) 3‐month functional outcome rate, (c) in‐hospital mortality rate, and (d) symptomatic intracranial hemorrhage (sICH) rate

Telestroke increased the odds of successful treatment within 3 hr by roughly twofold (Figure 4a). Furthermore, it was also associated with higher rate of functional outcome (Figure 4b) and lower in‐hospital mortality (Figure 4c). On the contrary, sICH rate was similar across arms (Figure 4d) with no heterogeneity observed (*I* ^2^ = 0%, *p* = .47). Except for 3‐month functional outcome rate—which yielded low heterogeneity (*I* ^2^ = 44%, *p* = .17), all outcomes showed no heterogeneity, as proven by *I* ^2^ value of 0%.

Assessment of publication bias through funnel plot was not conducted since no outcome yielded sufficient number of included studies (*n* < 10) (Higgins & Green, 2011).

## 4 DISCUSSION

The pooled data favored the implementation of telestroke as parts of prehospital and in‐hospital management of acute stroke patients in rural stroke networks. Both MSU and remote thrombolysis substantially reduced OTT, which subsequently allowed higher IVT rates in telestroke arms. In addition to shorter OTT and higher IVT rates, telestroke implementation also resulted in higher functional outcome as well as lower mortality rates.

Mobile stroke unit is an ambulance equipped with a point‐of‐care (POC) laboratory, a CT scanner, and telemedicine communication operated by a specialized stroke unit (Helwig et al., 2019; Walter et al., 2012). In a MSU‐incorporated stroke network, suspected stroke patients underwent anamnesis and neurological examinations. In addition, POC laboratory and imaging services were also performed and transmitted to in‐hospital stroke experts to perform triage, where suspected large vessel occlusion (LVO) and/or intracranial hemorrhage (ICH) patients were transferred to nearest CSC, while those without suspected LVO and/or ICH were given IVT directly at the emergency site or admitted to nearest PSC (Helwig et al., 2019).

The significant reduction of treatment times in MSU implementation was mainly attributed to shorter decision time and the obviated need for secondary transfers (Helwig et al., 2019; Walter et al., 2012). However, it should be noted that the substantial reduction of alarm‐to‐decision time as reported by Helwig et al. (2019) resulted from the significantly longer time required for vascular imaging. Nevertheless, Walter et al. (2012) confirmed this finding by emphasizing the superiority of MSU in terms of shortening diagnosis and treatment decision times. Furthermore, in the case of patients with LVO and eligible for thrombolytic therapies, IVT may be administered at the emergency site, thus extending the golden window for acute stroke care and allowing intra‐arterial reperfusion to take place, hence increasing favorable outcomes of those patients (Walter et al., 2010). This is supported by the fact that MSU significantly increased the number of patients treated with intra‐arterial recanalization by 33.3% while also reducing the time needed to perform this treatment modality by 42.3 min (Helwig et al., 2019).

Our findings confirmed the previous review by Mathur et al. (2019), stating that prehospital telestroke triage significantly reduced time required for treatment—evident in the reduction of secondary transfer rates and in‐hospital delays, thus increasing the proportion of patients treated within 3–4.5 hr. Furthermore, videoconferencing‐based remote neurological examinations have also showed significant improvements in acute stroke care, as stated by Hubert, Muller‐Barna, and Audebert (2014) The implementation of this approach is promising with current exponential technological and network developments (Hubert et al., 2014).

Currently, prehospital telestroke networks (i.e., mobile stroke units) have been established in 18 areas scattered over the world, where 12 networks are in the United States of America (USA). In addition, 12 networks may be established in the upcoming years (Walter et al., 2018). The abundant rise of these telestroke networks may call for further developments to expand their coverages to rural populations, thus may provide better evidences regarding the implementation of MSU in rural areas.

Contrary to MSUs which yield the concept of bringing hospitals to patients (Helwig et al., 2019), hub‐and‐spoke network models aim to bring specialized care to nonspecialized hospitals (Meyer et al., 2008). When acute stroke patients were presented to spoke emergency department, hub neurologists were contacted to perform teleconsultation (through telephone consultation and/or videoconference) and remote thrombolysis (Meyer et al., 2008). These enabled hub neurologists to perform real‐time clinical examination (if performed through videoconference) and review brain imaging through teleradiology—except for three studies(Frey et al., 2005; Mohr et al., 2019; Wang, Rose, Honings, Garwacki, & Milbrandt, 2000) where medical imaging was assessed by spoke radiologists rather than hub neurologists. This concept was shown to be efficacious and safe, as shown by shorter OTT, higher IVT rates, and lower mortality rates. Although our results did not show significant increase in IVT rates, robust model was obtained for increased proportion of patients treated within 3‐hr time, thus subsequently improved the proportion of patients with better outcomes. When analysis between telemedicine approaches was undertaken, videoconference and telephone‐only consultation yielded similar results—suggesting that both modalities are beneficial in resource‐limited settings in terms of increasing IVT rate and reducing OTT.

The results pooled in our study confirmed the association between telestroke and higher IVT rate (McDermott et al., 2019) and little to no difference on sICH rate (Baratloo et al., 2018; Kepplinger et al., 2016; Zhai et al., 2015). However, in contrast to previous meta‐analyses of telestroke implementation involving both rural and urban populations (Baratloo et al., 2018; Kepplinger et al., 2016; Zhai et al., 2015), our study discovered that telestroke significantly reduced in‐hospital mortality and 3‐month functional outcome rates although some of the pooled data were obtained from low‐to‐moderate quality of evidence. These findings suggest that the implementation of telestroke may possibly benefit rural inhabitants more than urban populations.

Although the clinical effectiveness of acute stroke management using telestroke is essential, cost‐effectiveness remains one of the main issues to ensure the applicability of telestroke. Dietrich et al. (2014) showed that MSU yielded benefit–cost ratios ranging from 2.16 at 43.01 km to 6.85 at 64.88 km. Additionally, MSU was expected to be cost‐efficient in a minimum population density of 79 inhabitants per km^2^, indicating its applicability in rural areas (Dietrich et al., 2014). Similar to MSU, hub‐and‐spoke network models were also cost‐effective in lifetime horizons. Although upfront costs are prominent, long‐term benefits of reduced disability gained from enhanced stroke care outweigh the initial costs (Bladin & Cadilhac, 2014; Nelson, Saltzman, Skalabrin, Demaerschalk, & Majersik, 2011). Possible burnout of neurologists might present another challenge to telestroke implementation. However, the opportunity to execute meaningful work by taking part in optimizing access to stroke care and mentoring regional physicians via telemedicine may actually increase professional satisfaction, thereby reducing burnout risk (Bagot, Cadilhac, Kim, Vu, & Bladin, 2017). Furthermore, training of regional non‐neurologist physicians would help to enhance their skills in making neurological diagnosis and taking appropriate management, thus alleviating the burden of neurologist shortfall (Freeman, Vatz, Griggs, & Pedley, 2013).

The favorable outcomes of telestroke emphasize the importance of implementing this novel approach in the management of rural stroke patients. Based on the results pooled in this study, the implementation of telestroke may significantly improve stroke's chain of survival, as depicted by the 8 D’s of stroke care framework (Jauch et al., 2013). This is especially true where prehospital telestroke may improve dispatch, delivery, and door by enabling rapid activation of EMS and transport of patient by EMS personnel, as well as prompt triage to appropriate stroke centers, respectively, while in‐hospital telestroke implementation may improve data, decision, and drug by shortening the amount of time needed to underwent diagnosis procedures as well as treatment decisions and administrations.

A study found that lack of proper infrastructure or road access in rural areas may significantly lengthen OTT time, thus contributing to the low rate of thrombolysis (Alasheev et al., 2017). Telemedicine helps to address this delay by allowing neurologists from stroke centers to perform quick assessment, triage, and give emergency treatment advices to EMS dispatchers even before patients’ arrival at the hospital (Jauch et al., 2013)—as shown by a reduction of roughly half an hour when compared to conventional EMS (Helwig et al., 2019). Quick and accurate triage by hub neurologists is also important as it helps paramedics to coordinate and send patients to appropriate hospital according to their needs, thus reducing the time wasted from unnecessary transfer (Jauch et al., 2013).

With regard to in‐hospital stroke management, telestroke allows the quick making of accurate treatment decisions for acute stroke patients arriving in EDs of rural hospitals where no neurologist is available, thus compensating for the lack of neurologists and human resources in these areas (Mathur et al., 2019). With regard to in‐hospital stroke management, telestroke allows the quick making of accurate treatment decisions for acute stroke patients arriving in EDs of rural hospitals where no neurologist is available, thus compensating for the lack of neurologists and human resources in these areas (Mathur et al., 2019). This is achieved by the utilizing videoconference and teleconsultations to stroke specialists in hub hospitals. In the end, the clinical effectiveness of telestroke depends on the rate of successful drug administrations (Jauch et al., 2013), which is observed higher in telemedicine arms than controls.

Despite the fact that our study showed favorable outcomes on telestroke usage, the lack of studies investigating prehospital management of acute stroke and the observed heterogeneity in OTT and IVT rate may limit the generalizability of our findings. The high heterogeneity of these outcomes (i.e., OTT and IVT) may be explained by the diverse rural geographical area and different telestroke technologies and approaches among studies; hence, indicating that interpretations should be carried out with caution. Furthermore, there were several studies reporting highly skewed outcomes; however, log transformation of the reported outcomes as per guideline (Higgins & Green, 2011) was not possible as some authors were unresponsive or unable to provide the requested data. To the extent of our knowledge, this is the first meta‐analysis conducted to analyze the effect of telestroke on resource‐limited settings. Although language bias may exist due to search limitations, this study involved a relatively large populations of 28,496 patients—emphasizing its representability. In addition, only one study (Ziegler et al., 2008) was excluded due to incomprehensible language, suggesting that any potential bias was negligible.

Furthermore, as most of the studies included in this study yielded moderate‐to‐serious risk of bias, further studies with higher quality of evidences are needed to confirm our findings (e.g., assigning concurrent control rather than historical control group). Although interpretations should be made with caution due to heteroscedastic effect sizes, our findings could be further implemented to construct a telestroke network system which integrates telemedicine in both rural prehospital and in‐hospital acute stroke management. The evidence provided has also proven the feasibility of such system, hence, highlighting the potential for investment in telestroke. We hope that our findings could encourage stakeholders to utilize telestroke in rural settings more rigorously. With an optimized emergency stroke approach, better stroke outcomes could be achieved, thereby aiding to alleviate stroke burdens in these regions.

## 5 CONCLUSIONS

In summary, although the implementation of telestroke as parts of prehospital and in‐hospital management of acute stroke care in resource‐limited settings is promising, further studies with better quality of evidences are needed to confirm these findings. Telestroke enables proper triage and guidance by distant specialists as well as allowing remote imaging assessment, teleconsultation, and remote thrombolysis, all of which may contribute to shorter treatment times and higher treatment rates as well as lower mortality rates.

## AUTHOR CONTRIBUTION

GL, JS, and DW contributed to project conceptualization, investigation, data curation, and writing of the original draft. GL and ISW contributed to methodology, formal analysis, and resources. GL contributed to visualization. GL, APP, SWN, and ISW contributed to validation, funding acquisition, as well as critical review and editing of the manuscript. All authors have read and approved of the final manuscript for publication.

## DISCLOSURES

None.

## CONFLICT OF INTEREST

None declared.

### Peer Review

The peer review history for this article is available at https://publons.com/publon/10.1002/brb3.1787.

## Supporting information

Supplementary Material

## Data Availability

The data that supports the findings of this study are available in the supplementary material of this article.
