# Supplementary material for: Telestroke strategies to enhance acute stroke management in rural settings: A systematic review and meta‐analysis
Source: Brain Behav. 2020 Aug 18;10(10):e01787. doi: 10.1002/brb3.1787 (PMC7559631; doi:10.1002/brb3.1787)
Supplement: Supplementary file 1 — Supplementary Material [file BRB3-10-e01787-s001.docx]

**SUPPLEMENTARY MATERIAL**

**
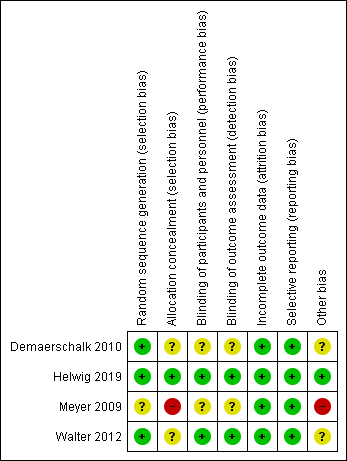
**

**Figure S1.** Risk of bias of included randomized studies using Revised Cochrane risk of bias tool for randomized trials (RoB 2)^1^


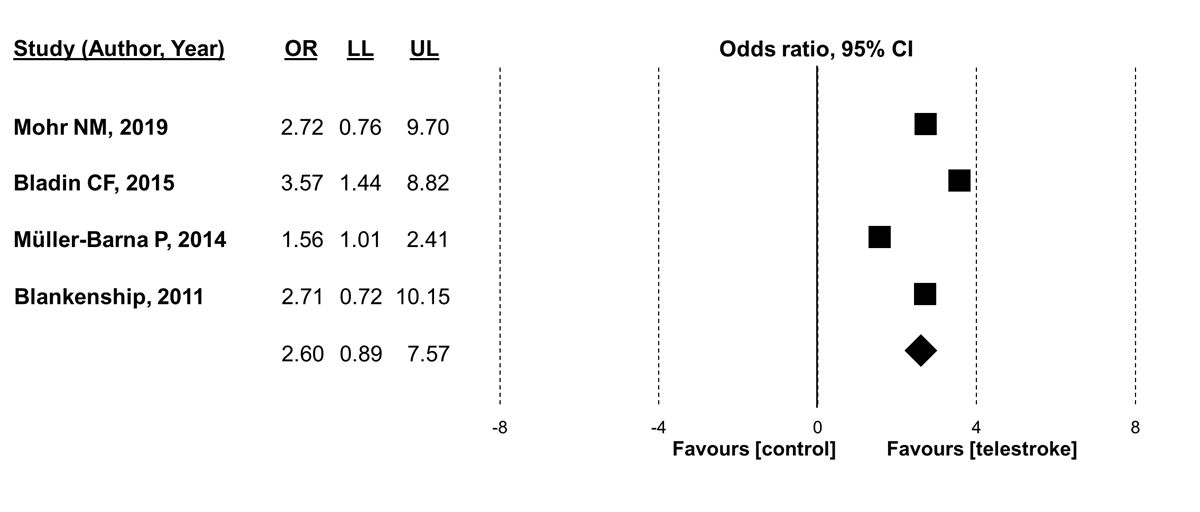


**Figure S2.** Forest plot showing leave-one-out sensitivity analysis of intravenous (IVT) rate. OR, odds ratio; LL, lower limit; UL, upper limit

**
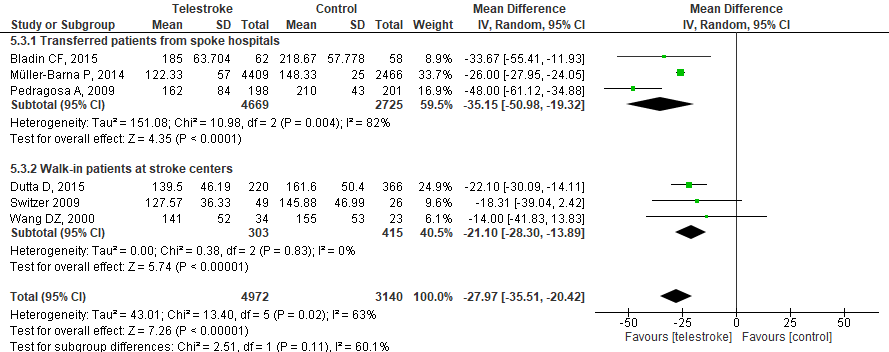
**

**Figure S3.** Forest plot showing the mean difference of onset-to-treatment time in subgroup analysis with respect to control groups


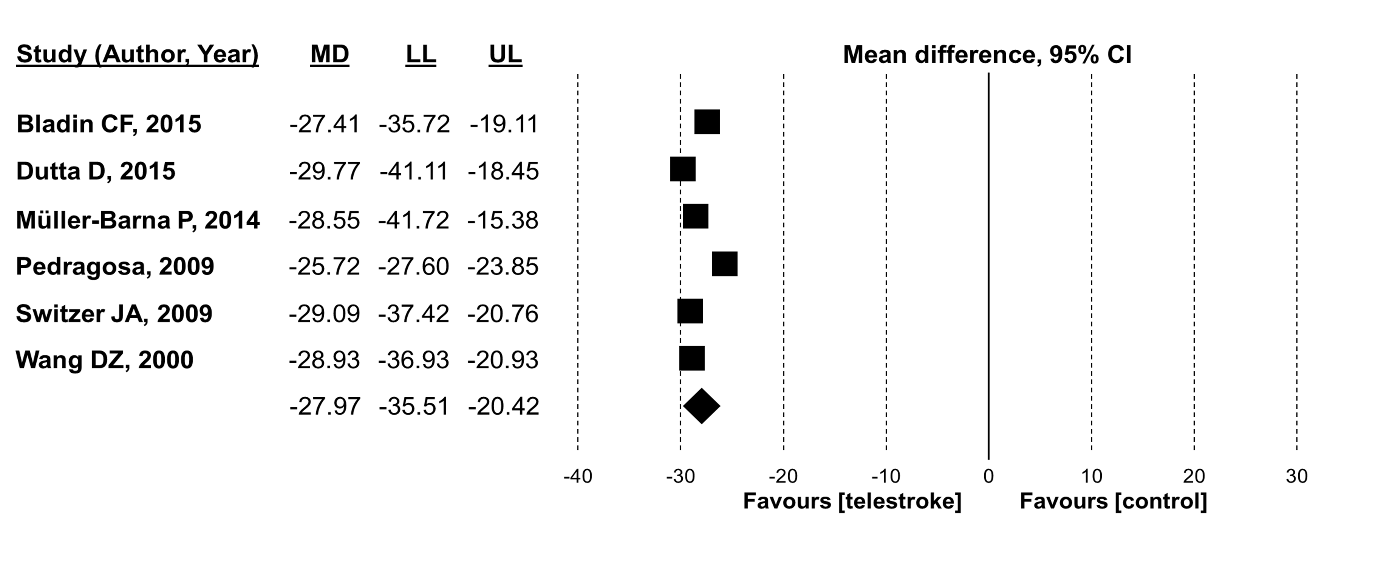


**Figure S4.** Forest plot showing leave-one-out sensitivity analysis of onset-to-treatment time (OTT). OR, odds ratio; LL, lower limit; UL, upper limit

**Table S1.** Literature search strategy

| **Database** | **Keywords** |
| --- | --- |
| PubMed | #44 telehealth[MeSH Terms]  #45 telemedicine[MeSH Terms]  #46 communities, rural[MeSH Terms]  #47 rural populations[MeSH Terms]  #48 health services, rural[MeSH Terms]  #49 rural hospitals[MeSH Terms]  #50 health, rural[MeSH Terms]  #51 remote consultation[MeSH Terms]  #52 acute stroke[MeSH Terms]  #53 cerebrovascular disorders[MeSH Terms]  #54 emergency[MeSH Terms]  #55 emergency care[MeSH Terms]  #56 emergency medical service[MeSH Terms]  #57 emergency care, prehospital[MeSH Terms]  #58 emergency hospital service[MeSH Terms]  #59 emergency health service[MeSH Terms]  #60 stroke[MeSH Terms]  #61 #44 OR #45 OR #51 OR tele*thrombolysis OR telestroke  #62 #46 OR #47 OR #48 OR #49 OR #50 OR non*urban OR rural OR remote area  #63 #52 OR #53 OR #60 OR cerebrovascular*  #64 #54 OR #55 OR #56 OR #57 OR #58 OR #59 OR pre*hospital OR acute OR emergency  #65 #61 AND #62 AND #63 AND #64 |
| CENTRAL | #1 MeSH descriptor: [Rural Health] explode all trees  #2 MeSH descriptor: [Rural Populations] explode all trees  #3 MeSH descriptor: [Hospitals, Rural] explode all trees  #4 MeSH descriptor: [Rural Health Services] explode all trees  #5 MeSH descriptor: [Stroke] explode all trees  #6 MeSH descriptor: [Cerebrovascular Disorders] explode all trees  #7 MeSH descriptor: [Telemedicine] explode all trees  #8 MeSH descriptor: [Emergencies] explode all trees  #9 MeSH descriptor: [Emergency Medical Services] explode all trees  #10 #1 OR #2 OR #3 OR #4 OR "non*urban" OR "remote area" OR "rural"  #11 #5 OR #6  #12 #7 OR "telehealth" OR "telestroke" OR "tele*thrombolysis"  #13 #8 OR #9 OR "prehospital" OR "acute"  #14 #10 AND #11 AND #12 AND #13 |
| Scopus  CINAHL  ProQuest | - ("stroke" OR "cerebrovascular") AND - ("telestroke" OR "telehealth" OR "telemedicine" OR “tele*thrombolysis”) AND - ("rural" OR "non*urban" OR "remote area") AND - ("pre*hospital" OR "acute” OR "emergency”) |
| Google Scholar | - ("stroke" OR "cerebrovascular") - ("telestroke" OR "telehealth" OR "telemedicine" OR “tele*thrombolysis”) - ("rural"[MeSH Terms] OR "non*urban" OR "remote area") - ("pre*hospital" OR "acute"[MeSH Terms] OR "emergency”[MeSH Terms]) |

**Table S2.** Risk of bias of included non-randomized studies using Risk of Bias in Non-randomized Studies of Interventions (ROBINS-I) tool

| **Study** | **Bias due to confounding** | **Bias in selection of participants into the study** | **Bias in classification of interventions** | **Bias due to deviations from intended interventions** | **Bias due to missing data** | **Bias in measurement of outcomes** | **Bias in selection of the reported result** | **Overall bias** |
| --- | --- | --- | --- | --- | --- | --- | --- | --- |
| Mohr NM (2019)^2^ | Moderate | Low | Low | Low | Low | Low | Low | Moderate |
| Bladin CF (2015)^3^ | Serious | Low | Low | No information | No information | Low | Low | Serious |
| Dutta D (2015)^4^ | Low | Low | Low | Low | Low | Low | Low | Low |
| Müller-Barna P (2014)^5^ | Serious | Low | Low | No information | No information | Low | Low | Serious |
| Dharmasaroja PA (2010)^6^ | Moderate | Moderate | Moderate | Low | No information | Moderate | Low | Moderate |
| Khan K (2010)^7^ | Serious | Low | Low | Moderate | Low | Low | Low | Serious |
| Pedragosa A (2009)^8^ | Serious | Low | Low | No information | No information | Low | Low | Serious |
| Switzer JA (2009)^9^ | Serious | Low | Low | Moderate | Moderate | Low | Low | Serious |
| Audebert HJ (2009)^10^ | Low | Low | Low | Moderate | Low | Low | Low | Moderate |
| Schwab S (2007)^11^ | Moderate | Low | Low | Low | Low | Low | Low | Moderate |
| Audebert HJ (2006)^12^ | Low | Low | Low | Low | Low | Low | Low | Low |
| Audebert HJ (2006)^13^ | Moderate | Low | Low | Moderate | Low | Low | Low | Moderate |
| Frey JL (2005)^14^ | Moderate | Low | Low | Moderate | No information | Low | Low | Moderate |
| Wiborg A (2003)^15^ | Moderate | Low | Low | Moderate | Moderate | Low | Low | Moderate |
| Wang DZ (2000)^16^ | Moderate | Low | Low | No information | No information | Low | Low | Moderate |

**Table S3.** Characteristics of included studies

| **Author (Year), Location** | **Study characteristics** | | | **Subject characteristics** | | | **Risk of bias**^†^ |
| --- | --- | --- | --- | --- | --- | --- | --- |
|  | **Study design (Recruitment period)** | **Settings** | **Intervention** | **Sample size** | **Mean age (years)** | **Male sex (%)** |  |
| **Prehospital settings** | | | | | | | |
| Helwig A (2019)^17^, Germany | Randomized, concurrent-controlled (Jun 2015 to Nov 2017) | Two non-urban CSCs and 8 PSCs with catchment areas of MSU of 16 min (mean distance to hospital, MSU vs control: 6.6 vs 4.0 km) | **I:** MSU consisting of videoconference consultations, neurological examinations, POCT, and teleradiology  **C:** Optimized pre-hospital management using LAMS stroke scale (Prehospital) | 116 | 74.5 | 37.9 | (-) |
| Walter S (2012)^18^, Germany | Randomized, concurrent-controlled (Nov 2008 to Jul 2011) | Single CSC in mixed urban-rural areas (mean distance to hospital, MSU vs control: 6 vs 8 km) | **I:** MSU consisting of videoconference consultations, neurological examination, POCT, teleradiology, and on-site remote thrombolysis  **C:** Usual care enhanced with POCT (In-hospital) | 100 | 71.5 | 63.0 | (?) |
| **In-hospital settings** | | | | | | | |
| Mohr NM (2019)^2^, USA | Non-randomized, concurrent-controlled (Nov 2007 to Aug 2015) | Out-of-town PSCs (hub) serving 19 federally designed rural critical access hospitals (spoke) | **I:** High-definition real-time videoconference between a local ED and a board-certified emergency physician with experienced ED nurse  **C:** Usual care when telestroke was not available and/or not utilized (In-hospital) | 638 | - | - | Moderate |
| Bladin CF (2015)^3^, Australia | Pre-post, historical-controlled (Jan to Dec 2010 for control, Oct 2011 to Oct 2012 for telestroke) | Metropolitan-based PSCs (hub) and a rural regional hospital serving >100,000 inhabitants | **I:** Videoconference between the regional hospital ED and a stroke specialist, teleradiology  **C:** Usual care before telestroke implementation (In-hospital) | 120 | 76.0 | 55.8 | Serious |
| Dutta D (2015)^4^, United Kingdom | Non-randomized, concurrent-controlled (Jan 2012 to Dec 2013) | Seven mixed urban-rural hospitals in southwest England (>2.25 million inhabitants) | **I:** Out-of-hours remote thrombolysis after telephone consultations between on-call remote stroke consultant and local in-house thrombolysis-certified clinician along with teleradiology  **C:** In-house thrombolysis in stroke centers (In-hospital) | 586 | 76 | 50.8 | Low |
| Müller-Barna P (2014)^5^, Germany | Pre-post, historical-controlled (2002 to 2012) | Two CSCs (hub) and 15 rural regional hospitals (spoke) covering an area of 17,887 km^2^ with 2.28 million inhabitants | **I:** Videoconference and telephone consultations as well as teleradiology transmission between on-site physician at spoke hospital and vascular neurologist at stroke centers, multidisciplinary stroke team with standardized stroke care protocols continually trained by multidisciplinary education team from two hub centers  **C:** Usual care before telestroke implementation (In-hospital) | 10,817 | 75.4 | 50.1 | Serious |
| Dharmasaroja PA (2010)^6^, Thailand | Non-randomized, concurrent-controlled (Jun 2007 to Feb 2008) | Single PSC (hub, 460 beds, a 64-slide CT, 4 neurosurgeons, 2 stroke neurologists) located 10-116 km (13-106 min) from 25 rural spoke hospitals | **I:** Telephone consultations between an on-duty neurology resident, an emergency room physician, and teleradiology (referred patients)  **C:** Walk-in patients receiving usual care (In-hospital) | 576 | 62 | 55.6 | Moderate |
| Khan K (2010)^7^, Canada | Non-randomized, historical-controlled (Mar 2007 to 2008) | Single PSC (hub) located 100-400 km from 7 rural spoke hospitals in an area of >1.5 million inhabitants | **Video arm:** Videoconference in four hospitals for direct consultation, remote neurological examination and teleradiology between ED physician and hub stroke neurologists  **Telephone arm:** Telephone-only consultations in three hospitals (In-hospital) | 44 | 68 | 54.5 | Serious |
| Demaerschalk BM (2010)^19^, USA | Randomized, concurrent-controlled (Dec 2007 to Oct 2008) | Multi-rural spoke hospitals located 299-300 km away from PSC (hub) | **Video arm:** Two-way audio/video consultations between hub vascular neurologist and spoke ED, remote neurological examination, and teleradiology  **Telephone arm:** Telephone-only consultations between hub consultant and spoke ED and teleradiology (In-hospital) | 54 | 66.3 | 50.0 | (?) |
| Meyer BC (2009)^20^, USA | Randomized, concurrent-controlled (Aug 2004 to Apr 2007) | Four rural spoke hospitals located 48-563 km away from PSC (hub) | **Video arm:** Two-way audio/high-definition video consultation between hub vascular neurologist and spoke ED, remote neurological examination, and teleradiology  **Telephone arm:** Telephone-only consultations between hub consultant and spoke ED and teleradiology (In-hospital) | 222 | 69.7 | 48.6 | (+) |
| Pedragosa A (2009)^8^, Spain | Pre-post, historical-controlled (2006 to 2007) | Single PSC (hub) located 70 km away from a rural spoke hospital | **I:** Videoconference between hub stroke experts and spoke physicians allowing remotely controlled camera (min. bandwidth 512 kbit/s, image res. 1024 x 768 px) to perform remote neurological consultation, teleconsultation, and teleradiology  **C:** Usual care involving patient transfer from spoke to hub hospitals (In-hospital) | 399 | 75 | - | Serious |
| Switzer JA (2009)^9^, USA | Non-randomized, historical-controlled (2005 to 2009) | Single PSC (hub) located 60-204 km away from 12 rural spoke hospitals | **I:** Web-based system allowing videoconference, teleconsultation and teleradiology between hub stroke specialists and spoke ED physician.  **C:** Patients presented directly to the hub receiving usual care (In-hospital) | 75 | 63 | 40 | Serious |
| Audebert HJ (2009)^10^, Germany | Non-randomized, concurrent-controlled (Jul 2003 to Mar 2005) | Two academic PSCs (hub) located up to 75 km away from 10 rural spoke hospitals | **I:** 24/7 videoconference link between hub neurologists and spoke ED allowing teleconsultation and teleradiology  **C:** Usual care in spoke hospitals involving patient transfer to hub hospitals | 3060 | - | - | Moderate |
| Schwab S (2007)^11^, Germany | Non-randomized, concurrent-controlled (Feb 2003 to Nov 2004) | Two academic PCSs (hub) and 12 rural spoke hospitals | **I:** Two-way videoconference between hub stroke experts and local ED enabling teleradiology and remote thrombolysis via videoconference  **C:** Consecutive stroke patients receiving thrombolysis at stroke centers (In-hospital) | 302 | 69.5 | 61.2 | Moderate |
| Audebert HJ (2006)^12^, Germany | Non-randomized, concurrent-controlled (Jul 2003 to Mar 2005) | Two academic PSCs (hub) and 12 rural spoke hospitals | **I:** High-speed videoconference (up to 2Mb/s) enabling teleconsultation, remote neurological examinations, and teleradiology  **C:** Conventional remote thrombolysis via usual care with elective conventional consultation with on-call neurologists (In-hospital) | 3122 | 72.8 | 49.6 | Low |
| Audebert HJ (2006)^13^, Germany | Non-randomized, concurrent-controlled (Jan to Dec 2004) | Two academic PSCs (hub) located up to 35 km away from 12 non-urban spoke hospitals | **I:** Real-time high-speed videoconference enabling teleconsultation, remote patient examination, and teleradiology, central organization of interhospital transfers, establishment of specialized stroke wards and teams receiving continuing trainings on standardized stroke care protocols  **C:** In-house thrombolysis in stroke centers (In-hospital) | 6616 | 69.7 | 59.6 | Moderate |
| Frey JL (2005)^14^, USA | Non-randomized, concurrent-controlled (1998 to 2002) | Single CSC (hub) and multi-rural spoke hospitals | **I:** Telephone consultation between emergency physicians and on-call stroke team allowing teleconsultation  **C:** In-house patients in stroke centers receiving usual care (In-hospital) | 126 | 63.9 | 54.76 | Moderate |
| Wiborg A (2003)^15^, Germany | Non-randomized, concurrent-controlled (Mar 2001 to Sep 2002) | Single PSC (hub) located 53-136 km ($\pm$80 min transport time) away from 7 rural spoke hospitals serving an area of 100-150 inhabitants/km^2^ | **I:** Videoconference (up to 384 kbit/s, 360 x 288 px) between local physician and stroke neurologists allowing teleconsultation, remote patient examination, and teleradiology  **C:** Usual care in spoke hospitals (In-hospital) | 623 | 67.5 | 50.3 | Moderate |
| Wang DZ (2000)^16^, USA | Non-randomized, concurrent-controlled (Jun 1996 to Dec 1998) | Single CSC (hub, 730 bed, 2 ambulance helicopter) and 20 rural spoke hospitals covering an area of $\pm$1.5 million inhabitants (14 hospitals serve towns with populations <20,000) | **I:** Telephone consultation between hub neurologist and local physician allowing teleconsultation  **C:** Walk-in patients in stroke center receiving usual care (In-hospital) | 900 | 71 | 57.4 | Moderate |

^†^Risk of bias of randomized studies were assessed using Revised Cochrane Risk of Bias Tool for Randomized Trials (RoB 2) and non-randomized studies using Risk of Bias in Non-randomized Studies of Intervention (ROBINS-I) tool; (-) low risk, (?) some concerns, (+), high risk. I, intervention group; C, control group; USA, United States of America; PSC, primary stroke center; CSC, comprehensive stroke center; MSU, mobile stroke unit; POCT, point-of-care testing; LAMS, Los Angeles Motor Scale; ED, emergency department.

**Table S4.** Outcomes reported in included studies (telestroke vs control)^†^

| **Author (Year)** | **IVT rate; n/N (%)** | **OTT (min)** | **In-hospital mortality; n/N (%)** | **Patients treated** $\boldsymbol{\leq}$**3 h; n/N (%)** | **3-months functional outcome rate; n/N (%)** | **sICH; n/N (%)** | **Miscellaneous** |
| --- | --- | --- | --- | --- | --- | --- | --- |
| Helwig A (2019)^17^ | 16/32 (50.0) vs 14/39 (35.9), p=0.22 | 66.55±72.26 vs 73.95±67.35 | NA | NA | NA | NA | - Call-to-FMC time: 10.3±3.6 vs 41.5±12.8 min (p<0.001) - Call-to-decision time: 47.6±9.0 vs 583.0±1427.0 min (p=0.009) - Call-to-thrombolysis time: 50.1±10.1 vs 84.9±30.2 min (p<0.001) - Call-to-intra-arterial therapy: 141±51.16 vs 183±84 min (p=0.46) - Intra-arterial therapy rate: 100% vs 66.7% - Triage accuracy: 100% vs 69.8% (p<0.001) - Sensitivity: 100% vs 35.3% - Specificity: 100% vs 86.1% - PPV: 100% vs 54.5% - NPV: 100% vs 73.8% - Patient transfer rates: 0% vs 41.2% |
| Mohr NM (2019)^2^ | 9/33 (27.3) vs 11/76 (14.5); aOR=3.49 [95% CI: 1.5, 8.2], p=0.11 | NA | NA | NA | NA | NA | - Door-to-CT time: 45 (34-62) vs 69 (45-98) min (p<0.01) - Door-to-treatment time: 71 (65–119) vs 108 (59–112) min (p=0.85) - Patient transfer rates: 81% vs 52.7% - Home discharge: 2/44 (4.5) vs 4/91 (4.4) |
| Bladin CF (2015)^3^ | 62/138 (44.9) vs 58/144 (40.3) | 185±63.70 vs 218.67±57.78, p=0.11 | 6/62 (10) vs 4/58 (7) | NA | NA | 1/16 (6) vs 0/10 (0) | - Door-to-FMC time: 13 (6-23) vs 13 (5-45) min, p=0.94 - Door-to-CT time: 34 (20-58) vs 63 (29-103) min - Door-to-treatment time: 85 (72–117) vs 101 (75–153) min (p=0.32) - Length of stay: 4 (2-6) vs 3 (1-6) days (p=0.34) - Patient transfer rates: 20% vs 67% - Home discharge: 21/62 (34) vs 14/58 (24) |
| Dutta D (2015)^4^ | NA | 161.6±50.7 vs 139.5±48.32, p<0.001 | 14/220 (6.4) vs  26/366 (7.1), p=0.8612 | NA | 101/220 (46) vs 169/399 (46.1) | 8/220 (3.6) vs 17/366 (4.6), p=0.7085 | - Patients thrombolysed out of hours: 93.6% vs 20.2% (p<0.001) - 3-months mortality rate: 15% vs 17.5% (p=0.5032) |
| Müller-Barna P (2014)^5^ | 685/4409 (15.5) vs 63/2466 (2.6), p<0.001 | 122.33±54.07 vs 148.33±25.93 p<0.001 | 145/4618 (3.1) vs 74/1469 (5.0), p<0.0001 | NA | NA | NA | - Onset-to-door time: 60 (45-79) vs 68 (48-105) min (p=0.63) - Door-to-needle time: 80 (68-101) vs 40 (29-59) min (p<0.001) - Length of stay: 6 (4-9) vs 9 (6-13) days (p<0.0001) - Patients treated <60 min: 543/675 (80) vs 15/57 (26) - Patient transfer rates: 7% vs 11.5% |
| Walter S (2012)^18^ | 12/53 (23) vs 8/47 (17) | 72 (53-108) vs 152 (135-209), p<0.0001 | 6/53 (11) vs 2/47 (4) | NA | NA | 3/53 (5.7%) vs 2/47 (4.3%) | - Median onset-to-CT time: 56 (43-103) vs 97 (74-156) min (p<0.0001) - Median onset-to-laboratory time: 51 (40-95) vs 99 (70-140) min (p<0.0001) - Median onset-to-decision time: 56 (43-103) vs 104 (80-156) min (p<0.0001) - Median call-to-decision time: 35 (31-39) vs 76 (63-94) min (p<0.0001) - Median call-to-treatment time: 38 (34-42) vs 73 (60-93) min (p<0.0001) - IV thrombolysis/intra-arterial therapy rate: 23% vs 23% (p=0.81) - Serious adverse events: 7/53 (13) vs 10/47 (21) |
| Dharmasaroja PA (2010)^6^ | 110/406 (27) vs 14/170 (8) | 136.6±39.6 vs 170.2±42.7, p<0.0001 | NA | NA | 30/62 (48.4) vs 23/55 (58.2) | NA | - Onset-to-door time: 119.9±40.7 vs 80.8±41.8 min (p<0.0001) - Door-to-treatment time: 49.1±24.2 vs 56.2±20.2 min (p=0.083) - Mortality rate of 11% (12/110) |
| Khan K (2010)^7^ | NA | 171±60.5 vs 179±36.25 p=0.68 | NA | NA | NA | NA | - Onset-to-door time: 92 (18-210) vs 102 (24-171) min (p=0.68) - IV thrombolysis rate of 21% - Reduced patient transfer rate by 92.5% - Patients treated $\leq$3 h: 32/44 (72.7%) - Door-to-treatment time: 82±38.75 vs 77±29.75 min (p=0.46) - Length of stay: 3 vs 7 days - 3-months mortality rate: 9/40 (22.5%) - 3-months favorable outcome rate: 16/40 (40%) |
| Demaerschalk BM (2010)^19^ | 8/27 (30) vs 8/27 (30), p>0.99 | 164.8±31.7 vs 170.5±17.2 (p=0.798) | NA | NA | 10/22 (46) vs 9/24 (38) | 1/27 (4) vs 0/27 (0), p>0.99 | - Onset-to-decision time: 188.2±138.2 vs 164.8±28.6 min (p=0.067) - Call-to-decision time: 58.3±18.0 vs 50.0±18.8 min (p=0.098) - Consent-to-decision time: 48.6±30.7 vs 43.7±33.4 min (p=0.325) - Door-to-decision time: 100.5±28.4 vs 90.7±27.9 min (p=0.115) - Door-to-treatment time: 61.65±45.22 vs 58.25±39.54 min - Decision-to-treatment time: 23.3±26.7 vs 16.3±9.0 min (0.874) - Correct treatment decision (87%): 85% videoconference vs 89% telephone (p>0.99) - Overall mortality: 4% vs 11% |
| Meyer BC (2009)^20^ | 31/110 (28) vs 25/111 (23), OR=1.3 [95% CI: 0.7, 2.5], p=0.33 | 157.2±37.3 vs 143.0±33.05 (p=0.137) | NA | NA | 9/30 (30) vs 8/25 (32), p=1.00 | 2/28 (7) vs 2/25 (8), OR 0.8 [95% CI: 0.1, 6.3], p=1.00 | - Onset-to-decision: 258.0±229.88 vs 230.6±222.42 min (p=0.067) - Call-to-decision: 64.71±29.06 vs 55.24±33.88 min (p=0.025) - Consent-to-decision: 32.04±17.34 vs 22.86±23.61 min (p<0.001) - Door-to-decision: 99.79±43.47 vs 95.51±64.09 min (p=0.198) - Door-to-treatment time: 54.91±54.83 vs 55.55±60.72 min - Decision-to-treatment: 10.03±9.75 vs 15.58±8.51 min (p=0.019) - Correct treatment decision: 98% videoconference vs 82% telephone (OR=10.9, [95% CI: 2.7-44.6], p<0.0001) - Overall mortality: 19% vs 13% |
| Pedragosa A (2009)^8^ | 19/198 (10) vs 9/201 (5), p=0.07 | 162±84 vs 210±43, p=0.05 | NA | 135/198 (68) vs 60/201 (30), p=0.04 | NA | 0/198 (0) vs 0/201 (0), p=1.00 | - Patients evaluated by a specialized neurologist: 38% vs 17% (p<0.01) - Patients treated at spoke: 63% vs 0% (p=0.001) - Patient transfer rates: 20% vs 51%, p=0.002 - Home discharge: 93/198 (47) vs 60/201 (30), p=0.57 |
| Switzer JA (2009) ^9^ | NA | 127.57±36.33 vs 145.88±46.99 | NA | NA | NA | NA | - Patients treated <90 min: 11/49 (22) vs 5/26 (19) - Patients treated <2 h: 26/49 (50) vs 9/26 (35) |
| Audebert HJ (2009)^10^ | NA | NA | NA | NA | NA | NA | - 12-months mortality rate: 22.7% vs 24.4% (OR 0.89, [95% CI: 0.75, 1.07], p=0.223) - 12-months poor outcome rate: 46.1% vs 55.5% (OR 0.65, [95% CI: 0.54, 0.78], p<0.001) - 30-months mortality rate: 33.3% vs 35.0% (OR 0.93, [95% CI: 0.78, 1.11], p=0.400) - 30-months poor outcome rate: 53.2% vs 58.4% (OR 0.82, [95% CI: 0.68, 0.98], p=0.031) - 3-months mortality rate: 15.1% vs 16.8% (aOR 0.93 [95% CI:0.74, 1.17]) - 1-year mortality rate: 22.7% vs 24.5% (aOR 0.98 [95% CI: 0.80, 1.19]) - 900-days mortality rate: 32.0% vs 34.5% (aOR 0.95 [95% CI: 0.79, 1.14]) |
| Schwab S (2007)^11^ | NA | 140.6 vs 143.6, p=0.451 | NA | 164/170 (96.5) vs 125/132 (94.7), p=0.387 | 65/170 (38) vs 34/132 (33.7), OR 1.7 [95% CI: 0.7, 2.0], p=0.258 | NA | - 3-months mortality rate: 11.2% vs 13.4% - 6-months favorable outcome rate: 39.5% vs 30.9% (p=0.095) - 6-months mortality rate: 85.8% vs 87.1% (p=0.448) |
| Audebert HJ (2006)^12^ | 80/1731 (5) vs 4/988 (0.4%), p<0.0001 | NA | 149/1863 (8) vs 114/1151 | NA | 1053/1880 (56) vs 489/1056 (46) | NA | - 3-months mortality rate: 17% vs 18% (OR 0.88 [95% CI: 0.73, 1.06, p=0.178) - Patient transfer rates: 13% vs 13% - Length of stay: 10.7±6 vs 11.9±6 days, p<0.0001 - Home discharge: 762/1971 (39) vs 435/1151 (38), p=0.001 |
| Audebert HJ (2006)^13^ | 115/4727 (2.4) vs 110/1889 (5.8), p<0.01 | 134±30 vs 135±38, p=0.81 | 4/114 (3.5) vs 5/111 (4.5), p=0.74 | 109/115 (94.8) vs 94/110 (85.5), p=0.02 | NA | 9/115 (3.5) vs 3/110 (2.7) | - Onset-to-door time: 64±26 vs 74±31 min (p<0.01) - Door-to-CT time: 17±9 vs 27±16 min (p<0.01) - Door-to-treatment time: 68±23 vs 61±23 min (p=0.03) - CT-to-treatment time: 51±22 vs 34±22 min (p<0.01) - Patients treated $\leq$90 min: 5.2% vs 9.6% (p=0.13) - Patients treated $\leq$2 h: 31.3% vs 30.0% (p=0.49) - 7-days mortality rate: 3.5% (4, [95% CI:1.0, 8.7]) vs 0.9% (1, [95% CI: 0.0, 5.0]) (p=0.37) - Length of stay: 10.3±4.4 vs 10.5±6.3 days, p=0.53 |
| Frey JL (2005)^14^ | NA | NA | 4/53 (7) vs 1/73 (1), p=0.08 | NA | NA | 1/53 (1.9) vs 0/73 (0) | - Home discharge: 16/53 (30) vs 41/73 (56), p=0.004 |
| Wiborg A (2003)^15^ | NA | NA | NA | 46/79 (58) vs 86/226 (39), p=0.001 | 25/64 (39) vs 95/235 (40.4) | NA | - Patients treated >24 h: 8% vs 16% (p=0.049) |
| Wang DZ (2000)^16^ | NA | 141±52 vs 155±53 | NA | NA | NA | 3/60 (5) | - Door-to-CT time: 33±19 vs 32±21 min - Length of stay: 5 (1-16) vs 4 (1-27) days - Home discharge: 31/57 (54) |

^†^Unless specified, data are presented in mean ± standard deviation (SD), median (IQR), or n/N (%). IVT, intravenous thrombolysis; OTT, onset-to-treatment time; sICH, symptomatic intracranial hemorrhage; OR, odds ratio; aOR, adjusted odds ratio; FMC, first medical contact; CT, computed tomography.

**Supplemental Table V.** GRADE assessment of quality of body evidence

| **Outcomes** | **Risk of bias** | **Inconsistency** | **Indirectness** | **Imprecision** | **Publication bias** | **Large effect** | **Plausible confounding** | **Dose response gradient** |
| --- | --- | --- | --- | --- | --- | --- | --- | --- |
| IVT rate | Very serious | Serious | Not serious | Very serious | Strongly suspected | Large | No | No |
| OTT | Serious | Not serious | Not serious | Serious | Strongly suspected | No | Would reduce demonstrated effect | No |
| Patients treated $\leq$3 h | Serious | Not serious | Not serious | Serious | Undetected | No | Would reduce demonstrated effect | No |
| In-hospital mortality | Serious | Not serious | Not serious | Serious | Undetected | No | Would reduce demonstrated effect | No |
| 3-month functional outcome rate | Not serious | Very serious | Serious | Serious | Undetected | No | Would reduce demonstrated effect | No |
| sICH | Serious | Not serious | Not serious | Very serious | Undetected | No | Would reduce demonstrated effect | No |

GRADE, Grading of Recommendations Assessment, Development and Evaluation; IVT, intravenous thrombolysis; OTT, onset-to-treatment time; sICH, symptomatic intracranial hemorrhage.

**Supplemental References**

1. Sterne J, Savović J, Page M, Elbers R, Blencowe N, Boutron I, et al. RoB 2: a revised tool for assessing risk of bias in randomised trials. BMJ. 2019;366:I4898.

2. Mohr NM, Young T, Harland KK, Skow B, Wittrock A, Bell A, et al. Telemedicine is associated with faster diagnostic imaging in stroke patients: a cohort study. Telemed J e-health. 2019 Feb;25(2):93–100.

3. Bladin CF, Moloczij N, Ermel S, Bagot KL, Kilkenny M, Vu M, et al. Victorian Stroke Telemedicine Project: implementation of a new model of translational stroke care for Australia. Intern Med J. 2015 Sep;45(9):951–6.

4. Dutta D, Kendall J, Holmes C, Murphy P, Black T, Whiting R, et al. Evaluation of a telephone advice system for remote intravenous thrombolysis in ischemic stroke data from a United Kingdom network. Stroke. 2015;46:867–9.

5. Muller-Barna P, Hubert GJ, Boy S, Bogdahn U, Wiedmann S, Heuschmann PU, et al. TeleStroke units serving as a model of care in rural areas: 10-year experience of the TeleMedical project for integrative stroke care. Stroke. 2014 Sep;45(9):2739–44.

6. Dharmasaroja PA, Muengtaweepongsa S, Kommarkg U. Implementation of telemedicine and stroke network in thrombolytic administration: comparison between walk-in and referred patients. Neurocrit Care. 2010 Aug;13(1):62–6.

7. Khan K, Shuaib A, Whittaker T, Saqqur M, Jeerakathil T, Butcher K, et al. Telestroke in Northern Alberta: a two year experience with remote hospitals. Can J Neurol Sci. 2016/09/23. 2010;37(6):808–13.

8. Pedragosa A, Alvarez-Sabin J, Molina CA, Sanclemente C, Martin MC, Alonso F, et al. Impact of a telemedicine system on acute stroke care in a community hospital. J Telemed Telecare. 2009;15(5):260–3.

9. Switzer JA, Hall C, Gross H, Waller J, Nichols FT, Wang S, et al. A web-based telestroke system facilitates rapid treatment of acute ischemic stroke patients in rural emergency departments. J Emerg Med. 2009 Jan;36(1):12–8.

10. Audebert HJ, Schultes K, Tietz V, Heuschmann PU, Bogdahn U, Haberl RL, et al. Long-term effects of specialized stroke care with telemedicine support in community hospitals on behalf of the Telemedical Project for Integrative Stroke Care (TEMPiS). Stroke. 2009 Mar;40(3):902–8.

11. Schwab S, Vatankhah B, Kukla C, Hauchwitz M, Bogdahn U, Furst A, et al. Long-term outcome after thrombolysis in telemedical stroke care. Neurology. 2007 Aug;69(9):898–903.

12. Audebert HJ, Schenkel J, Heuschmann PU, Bogdahn U, Haberl RL. Effects of the implementation of a telemedical stroke network: the Telemedic Pilot Project for Integrative Stroke Care (TEMPiS) in Bavaria, Germany. Lancet Neurol. 2006 Sep;5(9):742–8.

13. Audebert HJ, Kukla C, Vatankhah B, Gotzler B, Schenkel J, Hofer S, et al. Comparison of tissue plasminogen activator administration management between Telestroke Network hospitals and academic stroke centers: the Telemedical Pilot Project for Integrative Stroke Care in Bavaria/Germany. Stroke. 2006 Jul;37(7):1822–7.

14. Frey JL, Jahnke HK, Goslar PW, Partovi S, Flaster MS. tPA by telephone: extending the benefits of a comprehensive stroke center. Neurology. 2005 Jan;64(1):154–6.

15. Wiborg A, Widder B. Teleneurology to improve stroke care in rural areas: The Telemedicine in Stroke in Swabia (TESS) Project. Stroke. 2003 Dec;34(12):2951–6.

16. Wang DZ, Rose JA, Honings DS, Garwacki DJ, Milbrandt JC. Treating acute stroke patients with intravenous tPA. The OSF stroke network experience. Stroke. 2000 Jan;31(1):77–81.

17. Helwig SA, Ragoschke-Schumm A, Schwindling L, Kettner M, Roumia S, Kulikovski J, et al. Prehospital stroke management optimized by use of clinical scoring vs mobile stroke unit for triage of patients with stroke: a randomized clinical trial. JAMA Neurol. 2019 Sep;

18. Walter S, Kostopoulos P, Haass A, Keller I, Lesmeister M, Schlechtriemen T, et al. Diagnosis and treatment of patients with stroke in a mobile stroke unit versus in hospital: a randomised controlled trial. Lancet Neurol. 2012 May;11(5):397–404.

19. Demaerschalk BM, Bobrow BJ, Raman R, Kiernan T-EJ, Aguilar MI, Ingall TJ, et al. Stroke team remote evaluation using a digital observation camera in Arizona: the initial mayo clinic experience trial. Stroke. 2010 Jun;41(6):1251–8.

20. Meyer BC, Raman R, Hemmen T, Obler R, Zivin JA, Rao R, et al. Efficacy of site-independent telemedicine in the STRokE DOC trial: a randomised, blinded, prospective study. Lancet Neurol. 2008 Sep;7(9):787–95.
